# In vivo subcutaneous biocompatibility evaluation of decellularized tilapia fish skin in a rat model

**DOI:** 10.1038/s41598-025-21808-7

**Published:** 2025-10-30

**Authors:** Swathi Borra, Amrutha H. K., Vinita D’souza, Vaibhavi Shinde, Jagnoor Singh Sandhu, Arvind Kumar Pandey, Varadharajan Srinivasan, Raviraja N. Seetharam, Chaitanya Doshi, Ravindra Doshi, Kirthanashri S. V.

**Affiliations:** 1 Manipal Centre for Biotherapeutics Research, Manipal Academy of Higher Education, Karnataka, 576104 Manipal India; 2 Kore Additive Manufacturing and Medical Reconstruction Pvt Ltd, Mumbai, India; 3 Central Animal Research Facility, Kasturba Medical College, Manipal Academy of Higher Education, Karnataka, 576104 Manipal India; 4 Center for Animal Research, Ethics and Training (CARET), Manipal Academy of Higher Education, Karnataka, 576104 Manipal India; 5 Department of Anatomy, Kasturba Medical College, Manipal Academy of Higher Education, Karnataka, 576104 Manipal India; 6 Manipal Institute of Technology, Manipal Academy of Higher Education, Karnataka, 576104 Manipal India

**Keywords:** Decellularized tilapia fish skin (DTFS), Wound healing, Implant, Collagen, Biocompatibility, SDS-NaCl, Triton X-100-NaOH, Biological techniques, Biotechnology, Medical research

## Abstract

Decellularized tilapia fish skin (DTFS) has emerged as a promising biocompatible implant for wound healing due to its high collagen content, retained extracellular matrix (ECM) components, and structural similarity to human skin. This study investigates the decellularization of tilapia fish skin using two protocols including sodium dodecyl sulfate and sodium chloride (Treatment 1) and Triton X-100 and sodium hydroxide (Treatment 2) and evaluates their efficacy and biocompatibility. Decellularization efficiency was assessed through DNA quantification, histological analysis, and scanning electron microscopy. The DNA content in DTFS was significantly reduced (8.65 ± 1.25 ng/mg in Treatment 1 and 18.05 ± 0.85 ng/mg in Treatment 2) compared to native tilapia fish skin (286 ± 11 ng/mg), confirming effective removal of cellular material. Structural integrity of ECM was preserved in both treatments. In vivo biocompatibility was assessed by implanting the native and DTFS subcutaneously in Wistar rats, followed by hematological, biochemical (Urea, ALT, ALP, and LDH), and histological analysis of skin and liver tissues over 28 days. No significant abnormalities were observed in serum parameters or tissue morphology, indicating high biocompatibility. These results support the potential use of DTFS which are biocompatible as viable wound dressing for burn and chronic wound applications.

## Introduction

Tilapia refers to a group of freshwater fish within the family *Cichlidae*, comprising species from the *Oreochromis*, *Sarotherodon*, and *Tilapia* genera. Among these, *Oreochromis niloticus* (Nile tilapia) is the most widely cultivated, due to its rapid growth rate, high disease resistance, and environmental adaptability. These attributes have positioned tilapia as a cornerstone of aquaculture across nations with modest to moderate economic standings, offering an accessible and scalable protein source^1,2^. In India, tilapia aquaculture is rapidly expanding, with production reaching approximately 70,000 metric tons as of 2022. National targets under the Pradhan Mantri Matsya Sampada Yojana strive to enhance nationwide fish output, reaching 22 million metric tons by 2024-25, with tilapia playing a significant role. Beyond nutrition, tilapia skin has emerged as a promising biological material with antimicrobial, anti-inflammatory, and regenerative properties, particularly for wound healing applications^3^.

The biomedical interest in tilapia fish skin stems from its rich composition of type I collagen, glycosaminoglycans (GAGs), elastin, and tissue regeneration facilitators like EGF (epidermal growth factor), which are structurally and functionally analogous to components of human dermal tissue^4,5^. This composition supports its use as a natural, bioactive scaffold capable of promoting hemostasis, mitigating inflammation, and facilitating tissue regeneration. These features are especially desirable in burn and chronic wound management, where conventional dressings often fall short due to high costs, limited bioactivity, or suboptimal biocompatibility^7^.

Notably, clinical research, such as a Phase III randomized controlled trial conducted in Brazil, has demonstrated that glycerol-preserved tilapia skin accelerates re-epithelialization and improves patient comfort when compared to silver sulfadiazine, the current standard in burn care^8^. Antimicrobial peptides naturally present in the skin also provide innate protection against common pathogens such as *Staphylococcus aureus* and *Pseudomonas aeruginosa*^9^. Furthermore, both in vitro and in vivo studies have highlighted that low immunogenicity and favourable safety profile of tilapia collagen scaffolds, enhancing their appeal for clinical translation^10^.

However, despite these promising findings, robust preclinical evaluation of tilapia skin-based scaffolds particularly with regard to decellularization efficiency, ECM preservation, and host immune response remains limited^11^. Decellularization is a pivotal step in rendering xenogeneic tissues safe for implantation by eliminating cellular antigens while retaining the extracellular matrix (ECM). DNA quantification (< 50 mg/mg), Agarose gel electrophoresis (Showing no bands) and H&E staining (Absence of nuclear material) are recognized as gold standard methods for assessing decellularization. Successful validation through these tests indicates effective cellular removal, and adverse cell and host responses have been avoided^12^.

The effectiveness of this process directly influences implant biocompatibility, immune response, and integration potential. Existing protocols often differ in detergent type, ionic strength, and exposure duration, each impacting the balance between decellularization efficacy and matrix integrity^13^.

Globally, interest in fish-skin-derived dressings has led to the commercialization of cod- and tilapia-based products, such as Kerecis^®^ Omega3 and Pisciskin^®^, which are now positioned as sustainable alternatives to mammalian xenografts^14^. In India, research groups and startups are developing scalable, cost-effective methods for processing tilapia skin adhering to Good Manufacturing Practices (GMP), supported by regulatory frameworks from the Central Drugs Standard Control Organization (CDSCO) and Indian Council of Medical Research (ICMR)^15,16^. As per the Medical Devices Rules (2017), such products fall within the Class C or D devices, requiring rigorous biocompatibility, toxicity, and animal testing prior to approval for clinical use.

In addition to regulatory advancement, innovations such as growth factor incorporation, stem cell seeding, and 3D bioprinting are expanding the utility of fish-derived ECM scaffolds. These advancements are especially relevant to chronic wound care and diabetic ulcer management, where enhanced angiogenesis and tissue remodeling are critical^17^. As such, tilapia skin is no longer viewed merely as a low-cost alternative, but as a versatile therapeutic platform for bioengineered skin substitutes.

Various physical methods (e.g., freeze–thaw cycles, sonication, agitation), chemical agents (such as hydrogen peroxide and ethanol), and enzymatic treatments (including Trypsin-EDTA, DNase, and RNase) have been used in earlier research for decellularizing tilapia skin^18–20^. We employed two distinct chemical treatment protocols for the decellularization of tilapia fish skin. **Treatment 1** involves the use of **Sodium Dodecyl Sulfate (SDS)** and **Sodium Chloride (NaCl)**, while **Treatment 2** utilizes **Triton X-100** and **Sodium Hydroxide (NaOH)**. Notably, the pre-treatment and post-treatment procedures described in our methodology differ from those reported in previous studies^28–37^.**Treatment 1** introduces a novel chemical composition of only SDS and NaCl for decellularization, a formulation not previously reported, thereby enhancing the uniqueness of this method. In **Treatment 2**, prior to decellularization, tilapia fish skin is gamma-sterilized at 6–13 kGy for 8 h, followed by separate washes with 1X PBS and distilled water, which can improve the overall efficacy of the protocol. both treatments include post-washing step (1XPBS & Distilled water) and treatment with **Gibco™ Antibiotic-Antimycotic**, followed by **UV sterilization** for 30 min on each side of DTFS. These treatments further contribute to the distinctiveness and effectiveness of the decellularization approach.

**Fig. 1:**
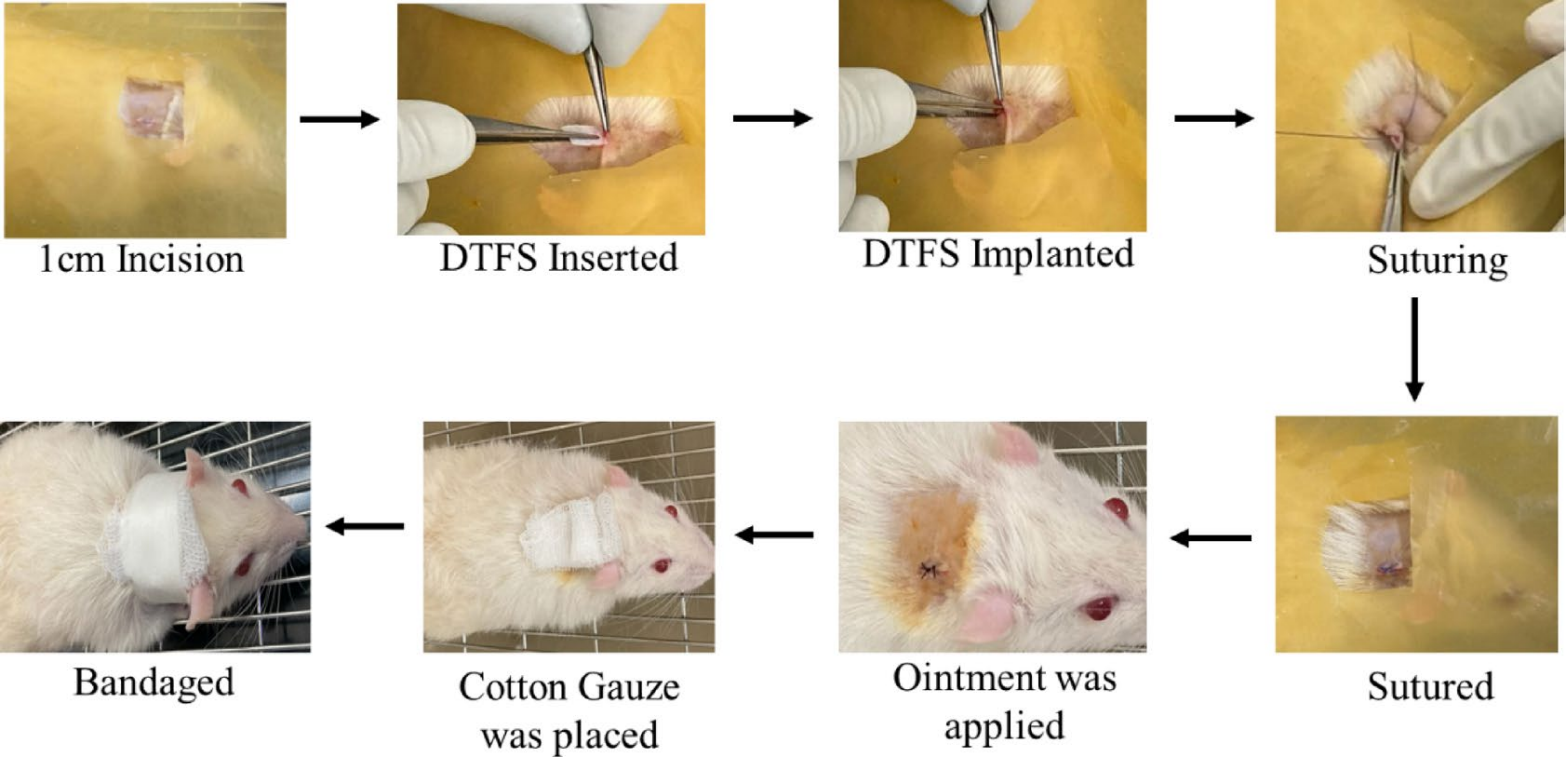
Pictorial representation of subcutaneous implantation on male Wistar rats.

Also, a wide range of decellularization methods was explored, as detailed in Fig. 1D. After careful evaluation, treatments 1 and 2 were selected for further analysis because of their remarkable efficacy in effectively removing cellular components. Notably, treatment 1 **(Patent Application No. 202441096704)** and treatment 2 **(Patent Application No.202541044101)** are our innovative techniques, which have been filed for an Indian patent, highlighting their uniqueness and potential applications in the field.

In this study, we systematically evaluate the efficacy of two decellularization protocols Treatment 1 (sodium dodecyl sulfate and sodium chloride) and Treatment 2 (Triton X-100 and sodium hydroxide), for generating biocompatible decellularized tilapia fish skin (DTFS). The comparative analysis includes DNA quantification, histological (H&E) staining, and scanning electron microscopy to assess ECM integrity and cellular clearance. Further, the in vivo biocompatibility of DTFS is examined using a Wistar rat model through hematological profiling, serum biochemical assays (ALT, ALP, LDH, urea), and histopathological evaluation of skin and liver tissues over a 28-day period. These findings aim to validate DTFS as a biologically active, safe, and cost-effective for wound healing, aligned with the goals of **Sustainable Development Goals (SDG) 3 and 12**.

## Materials and methods

### Materials

All reagents used were of molecular biology grade, with a minimum purity of 98.5%. Sodium dodecyl sulfate (SDS), Triton X-100, phenol, chloroform, isoamyl alcohol, and isopropanol were purchased from Sisco Research Laboratories, India. Proteinase K (25530015) was obtained from Invitrogen, India. Sodium chloride (NaCl) and sodium hydroxide (NaOH) were purchased from Qualigens, India, and sodium acetate was sourced from Tokyo Chemical Industry, India.

### Pre-treatment of tilapia skin

Tilapia (*Oreochromis niloticus*) was procured from local vendors in Mumbai, India. The skin was dissected, thoroughly washed with tap water, and stirred in distilled water at room temperature (pH ~ 7.0) for 10 min to remove scales. This step was repeated twice until fully descaled. The cleaned skin samples were lyophilized for 24 h and sterilized by gamma irradiation at a dose of 6–13 kGy in accordance with ISO 11,137 guidelines^21^. The sterilized fish skin was vacuum-packed at Kore Additive Manufacturing and Medical Reconstruction Pvt. Ltd., Mumbai, and shipped to Manipal Centre for Biotherapeutics Research (MCBR), Manipal, India.

#### Decellularization: treatment 1(patent application no: 202441096704)

Lyophilized fish skin was sectioned into 2 × 2 cm patches and immersed in 20% NaCl solution (pH 7.0) with stirring for 3 h at room temperature. This was followed by rinsing in distilled water (pH 7.0) for 2 h and subsequent treatment with 1% SDS (pH 6.5) for 3 h. The tissue was then washed with autoclaved 1X Phosphate Buffer Saline [P4474-1 L] (pH 7.4) for 1 h and finally rinsed in sterile distilled water for 1 h. All steps were conducted on a magnetic stirrer at 200 rpm. DTFS were stored at − 20 °C until further use.

#### Decellularization: treatment 2 (patent application no.202541044101)

Lyophilized fish skin (2 × 2 cm) was immersed in 0.1% Triton X-100 and stirred at for 16 h, followed by treatment with 0.1 N NaOH for 6 h. Samples were then washed sequentially with sterile 1X PBS (1 h) and autoclaved distilled water (1 h), both at room temperature. All treatments were performed at 200 rpm. The obtained DTFS were stored at − 20 °C.

#### Final sterilization

Native (non-decellularized) and DTFS were soaked in Gibco™ Antibiotic-Antimycotic (100X) [15240-062] solution for 30 min and subsequently UV-sterilized at 254 nm for 20 min on each side.

### Implant characterization

#### DNA quantification

DNA was extracted using a modified phenol–chloroform–isoamyl alcohol method. Briefly, 100 mg tissue from each group (native, Treatment 1, and Treatment 2) was lysed using Tris-EDTA buffer (TE-Buffer) and Proteinase K, incubated at 55 °C for 3 h. DNA was purified using sequential organic extraction, ethanol precipitation, and air drying. The DNA pellet was resuspended in TE buffer, and yield was quantified using a multimode microplate reader (Agilent Technologies, USA). Integrity was confirmed using 1% agarose gel electrophoresis^22^.

#### Hematoxylin and Eosin (H&E) staining

Lyophilised native and DTFS samples were fixed in 10% formalin, embedded in paraffin, sectioned in a sagittal plane (5 μm), dehydrated, and stained with hematoxylin and eosin^23^. Slides were imaged using an EVOS™ FL Auto 2 Imaging System (Invitrogen, USA).

#### Scanning electron microscopy

Lyophilized Native and DTFS samples were placed in a sagittal plane and gold-coated using a Quorum SC7620 sputter coater under vacuum (10 Pa for 120 s) and analyzed using Zeiss EVO MA18 SEM with Oxford EDS (Germany) to assess surface morphology and ECM architecture^6^.

#### SDS-Page

Proteins from native and DTFS samples were extracted by enzymatic digestion using acetic acid-pepsin solution for 24 h. Following digestion, the pH was adjusted to 7.4, and protein quantification was carried out using the Thermo Fisher Scientific BCA Protein Assay Kit (absorbance measured at 562 nm, Catalog No: [23227]) in accordance with the manufacturer’s instructions. About 30 µg/µL of digested samples of native and DTFS (Treatment 1&2) were run on 8% SDS-PAGE gel under constant voltage (100 V) for 90 min^39^. The gel was subsequently stained with Coomassie Brilliant Blue, destained, and visualized using an Invitrogen imaging system.

### Cell viability assay

NIH3T3 cells (Mouse fibroblast) were seeded in 96-well plates at a density of 1 × 10^4^ cells per well and allowed to adhere and attain confluency. Subsequently, the cells were treated with varying concentrations of digested samples of native (2, 4, 6, 8, 10 µg) and DTFS [Treatment 1(2, 4, 6, 8, 10 µg) & Treatment 2(0.2, 0.5, 0.7, 1, 2, 4, 6, 8, 10 µg)] extracts and incubated for 72 h. Following treatment, MTT solution [Himedia MTT cell culture tested, Catalog No: TC191] was added to each well and incubated for 4 hours to facilitate the formation of formazan crystals^40^. The medium containing MTT was then carefully removed, and the crystals were solubilized by adding DMSO, followed by incubation for 30 min, and the absorbance was measured at 570 nm.

### In vivo biocompatibility evaluation

#### Animal ethics approval

Animals used in this study were sourced from the Central Animal Research Facility (CARF), MAHE, Manipal. All animal procedures were conducted in accordance with the ARRIVE guidelines and approved by the Institutional Animal Ethics Committee (IAEC), Manipal Academy of Higher Education, Manipal (Approval No: IAEC/MCBR/45/2024), under CPCSEA guidelines (Registration No.94/PO/RReBi/S/99/CPCSEA). All methods were carried out in accordance with relevant guidelines and regulations.

#### Study design and surgery

Thirty healthy male Wistar rats (8–12 weeks old) were randomly divided into five groups (*n* = 6/group): Control (untreated rats), Sham (incision + suture), Native (rats implanted with native [non-decellularized] tilapia fish skin), Treatment 1(rats implanted with DTFS as per treatment 1) and Treatment 2 (rats implanted with DTFS as per treatment 2). Each group was further divided into two time points as day 14 and day 28. Under ketamine (100 mg/kg) and xylazine (20 mg/kg) anaesthesia, Hair on dorsal area was shaved and cleaned with 70% ethanol, followed by 1 cm dorsal skin incision and a skin pocket was made in deep fascia region, and the samples according to respective groups with size of 0.5cm^2^ were implanted in the subcutaneous pocket. Using absorbable sutures, the site was sutured and covered with mupirocin-infused gauze as shown in Fig. 1. Post-operative analgesics were administered. At designated time points, blood was collected via retro-orbital puncture, and rats were humanely euthanized by CO₂ asphyxiation in accordance with CPCSEA guidelines.

### Hematological analysis

Whole blood samples were analysed using a Celltac hematology analyser (Nihon Kohden, Japan) for total and differential counts, RBCs, WBCs, lymphocytes, and platelets.

### Biochemical analysis

Serum was separated by centrifugation (2,000 g, 15 min, 4 °C) and stored at − 80 °C. The following assays were performed according to manufacturers’ protocols: Urea: Elabscience Urea Colorimetric Assay Kit (absorbance at 580 nm)[Cat No.E-BC-K183-M]; ALT: Abcam ALT Assay Kit (absorbance at 580 and 570 nm)[Cat No.b241035]; ALP: Abcam ALP Assay Kit (absorbance at 405 nm)[Cat No.ab83369] and LDH: Elabscience LDH Assay Kit (absorbance at 450 nm)[E-BC-K046-M].

### Histological examination of organs

Post-mortem, rat liver and skin tissues were fixed in 10% formalin, embedded in paraffin, sectioned (5 μm), and stained using hematoxylin and eosin^24^. Slides were examined for tissue architecture and inflammatory changes using an EVOS™ imaging system. Histomorphic analysis of rat epidermis was carried out using freely downloadable ImageJ software [Java 1.8.0_345, 64-bit] by measuring epidermal thickness from H&E-stained skin sections across the different experimental groups.

### Statistical analysis

Statistical analyses were performed using GraphPad Prism v8.0.1 (GraphPad Software, USA). All values are presented as mean ± standard deviation (SD). One-way ANOVA with Tukey’s post hoc test was used for intergroup comparisons. A p-value < 0.05 was considered statistically significant^25^.

## Results

### DNA quantification

**Fig. 2:**
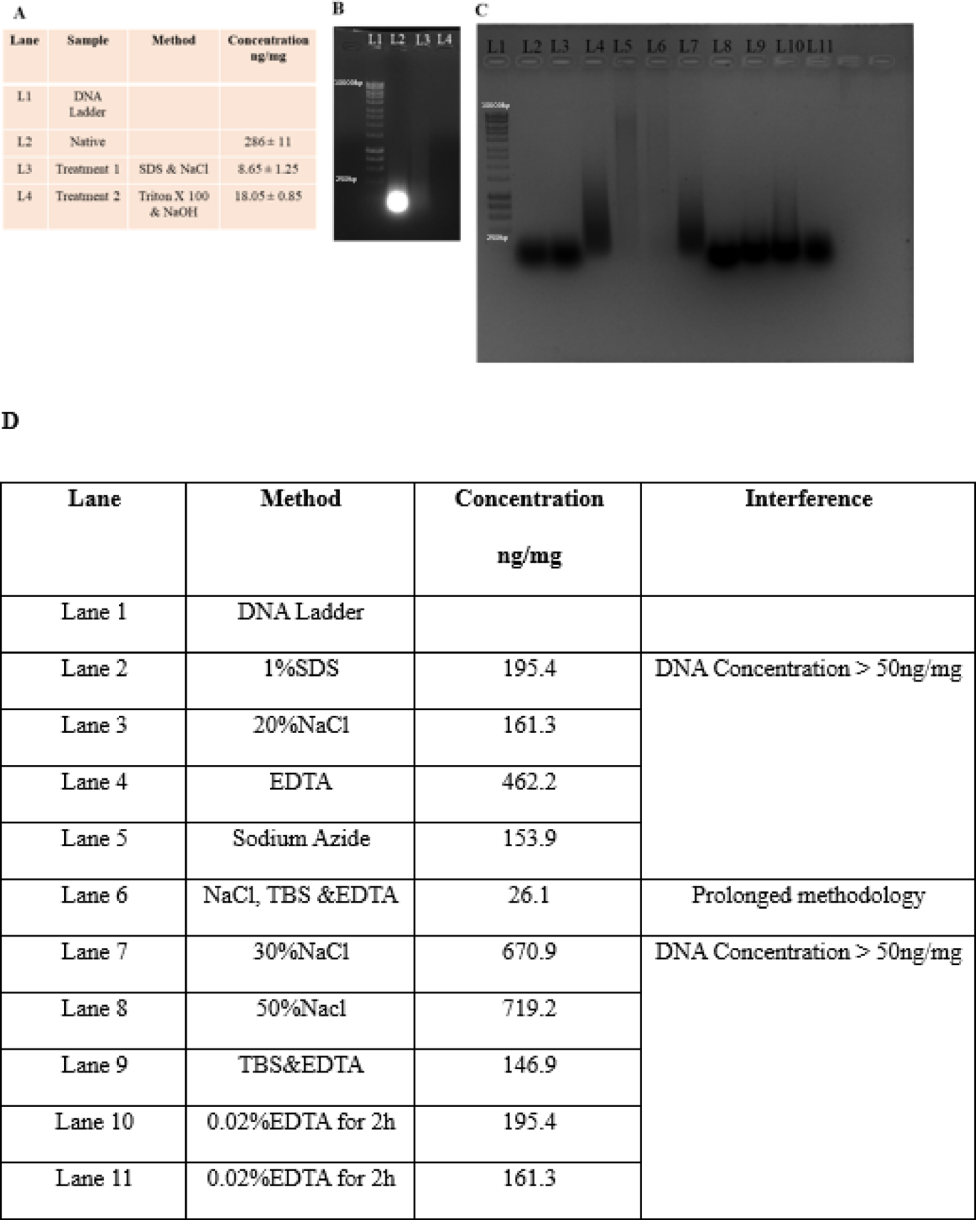
(**A**) The DNA quantification of native (non-decellularized) tilapia fish skin and DTFS, (**B**)The 1% agarose gel showed a clear DNA smear for the native group and no clear bands/smears were found in the decellularized groups, (**C**) 1% agarose gel of various decellularization methods as shown in (**D**).

The DNA concentration in native (non-decellularized) tilapia fish skin was 286 ± 11 ng/mg, significantly higher compared to the treated groups. Treatment 1 (8.65 ± 1.25 ng/mg) and Treatment 2 (18.05 ± 0.85 ng/mg), respectively (*p* < 0.001) (Fig. 2). Agarose gel electrophoresis further confirmed effective decellularization, native samples demonstrated strong DNA smears, whereas treatment groups showed no visible bands, indicating substantial removal of nucleic material. Also, multiple decellularization methods, as outlined in Table 1, were implemented; however, they proved ineffective in eliminating nuclear content, with DNA concentrations exceeding 50 ng/mg being reported.

**Table 1:** Various decellularization methods for tilapia fish skin from the literature.

	Author(s)	Treatment method	Residual DNA content	References
1	Lau et al.	SDS + Nuclease enzymes	~ 99.6% DNA removed (~ 1.8 ± 0.9 ng/mg)	^29^
2	Liu et al.	SDS + enzymatic treatment (for tendon scaffolds)	Significant reduction (dsDNA assay); exact value not given	^30^
3	Esmaeili et al.	NaOH + Triton X-100 + Freeze–thaw cycles	1.4 ± 0.7 ng/mg dry weight	^31^
4	Huang et al.	NaOH (0.5 M) + SDS (1%) + Nuclease	38.78 ± 5.84 ng/mg dry weight	^32^
5	Ibrahim et al.	Chemical sterilization of tilapia skin xenograft	< 50 ng/mg (qualitative, sufficient for medical use)	^33^
6	Garrity et al.	Tilapia skin hydrogel (decellularized, exact method not specified)	Not reported	^34^
7	Luo et al.	Gelatin hydrogel from tilapia + stromal vascular fraction	Not reported	^35^
8	Zhang et al.	0.5% Triton X-100 + 1% SDS (ionic detergents)	2.84% of native DNA remained	^36^
9	Huang et al.	Fresh tilapia skin + chemical decellularization	32.94 ng/mg dry weight	^37^
10	We et al.	Hypertonic/hypotonic cycles + Triton X-100	Qualitative only – “minimal genetic material”	^38^

### Hematoxylin and eosin (H&E) staining

**Fig. 3:**
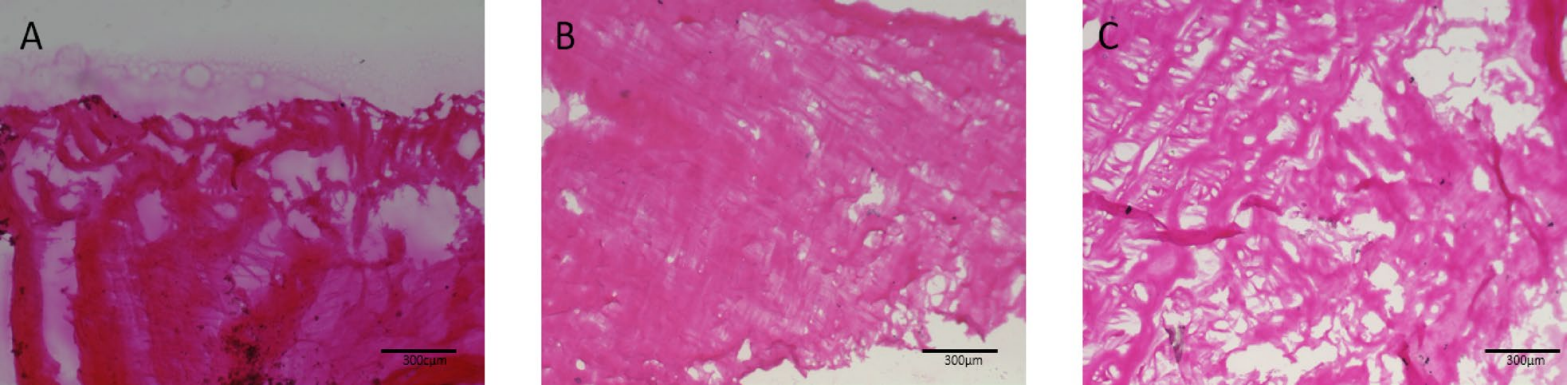
Histological sections of the native (non - decellularized) tilapia fish skin (**A**) and DTFS [**B**(Treatment 1) & C(Treatment 2)] at 10X magnification.

Histological analysis using hematoxylin and eosin staining was conducted to assess the structural integrity and cellular content of native and DTFS. As shown in Fig. 3, native fish skin (Fig. 3A) displayed a dense crisscross arrangement of collagen fibers and intact nuclei distributed throughout the tissue. In contrast, Treatment 1 & 2 (Fig. 3B&C) exhibited complete removal of cellular and nuclear components, with no visible nuclei observed. Significant collagen fiber architecture was retained in both treatment groups, indicating preservation of the extracellular matrix (ECM) during the decellularization process.

### Scanning electron microscopy (SEM)

**Fig. 4:**
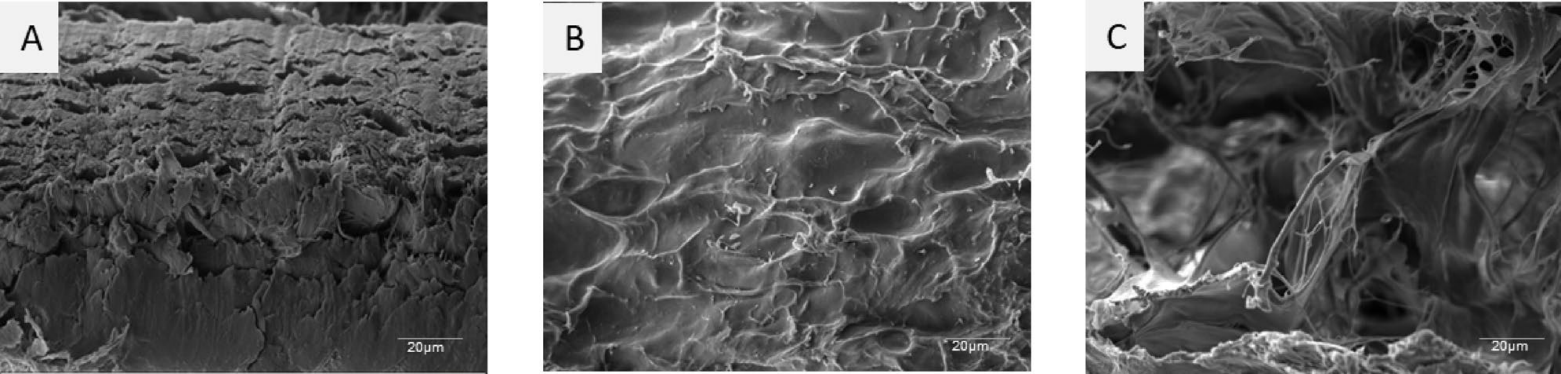
Scanning electron microscopy of native(A) and decellularized tilapia fish skin [B(Treatment 1) & C(Treatment 2)] at 1000X.

SEM imaging was performed to visualize the microstructural differences between native and DTFS. As shown in Fig. 4, the native fish skin (Fig. 4A) exhibited a compact, stratified architecture with dense tissue layers and minimal porosity. In contrast, Treatment 1 (Fig. 4B) revealed a more porous, wavy fibrillar arrangement with exposed collagen fibers and visible interfibrillar gaps, indicating disruption of cellular layers and partial opening of the extracellular matrix (ECM) network. Treatment 2 (Fig. 4C) displayed a highly porous and fibrillar microstructure, with evident fiber delamination and disordered ECM topography. The implant appeared less compact than Treatment 1, suggesting a greater degree of structural loosening and matrix expansion due to the milder decellularization process.

### SDS-Page

**Fig. 5:**
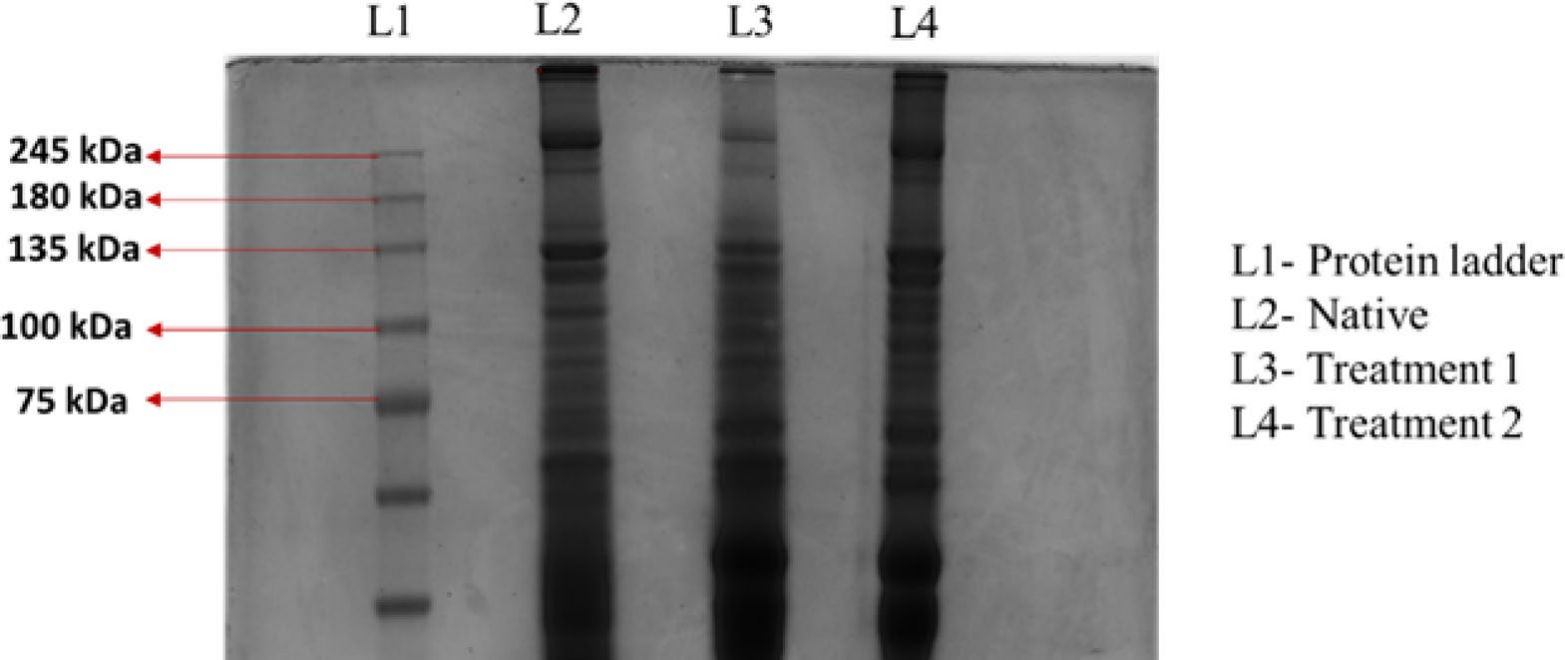
SDS -PAGE of Native and DTFS (Treatment 1&2).

SDS-PAGE analysis was carried out to assess the molecular weight distribution of proteins in the digested native and DTFS. Figure 5 shows the resulting protein profiles depicted as clear bands in both sample types. The presence of these bands in treatments 1&2 confirms that collagen was retained within the ECM following decellularization. Distinct protein bands corresponding to the α1 and α2 chains of type I collagen were observed at approximately 180& 135 kDa, while the β chain appeared at around 245 kDa. The 75 kDa band likely represents collagen degradation fragments or non-collagenous extracellular matrix proteins commonly retained after decellularization.

### Cell viability assay

**Fig. 6:**
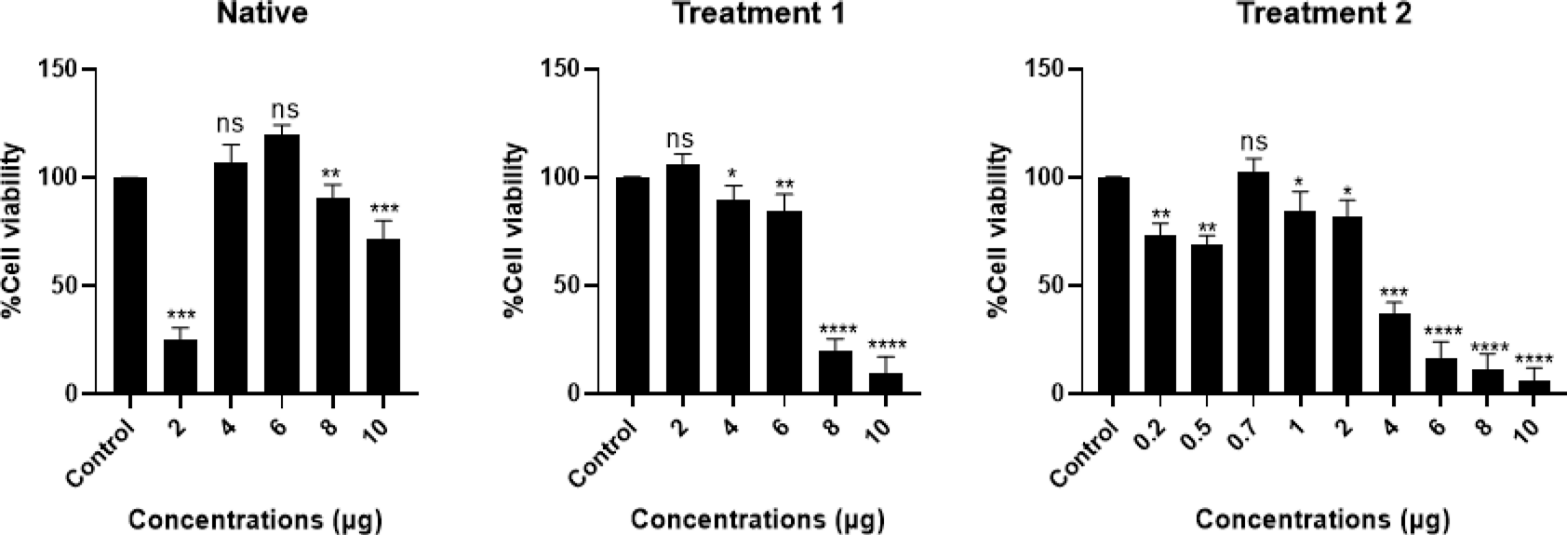
MTT assay of Native, Treatment 1and 2. Control (Complete Media),100% cell viability was observed at 6 µg for the native, 2 µg for Treatment 1, and 0.7 µg for Treatment 2. (*p < 0.05.**p < 0.01, ***p < 0.001, ****p < 0.0001).

A cell viability assay was conducted to evaluate the in vitro biocompatibility of native and DTFS. The native exhibited nearly 100% cell viability at a concentration of 6 µg when compared to the control group (complete media), as shown in Fig. 6. Similarly, DTFS treatments demonstrated high levels of cell viability, with treatment 1 showing cell viability at 2 µg and treatment 2 maintaining cell viability at 0.7 µg compared with the control. These findings suggest that both native and DTFS support cellular proliferation and survival, thereby confirming in vitro biocompatibility.

### Hematological profile

**Fig. 7:**
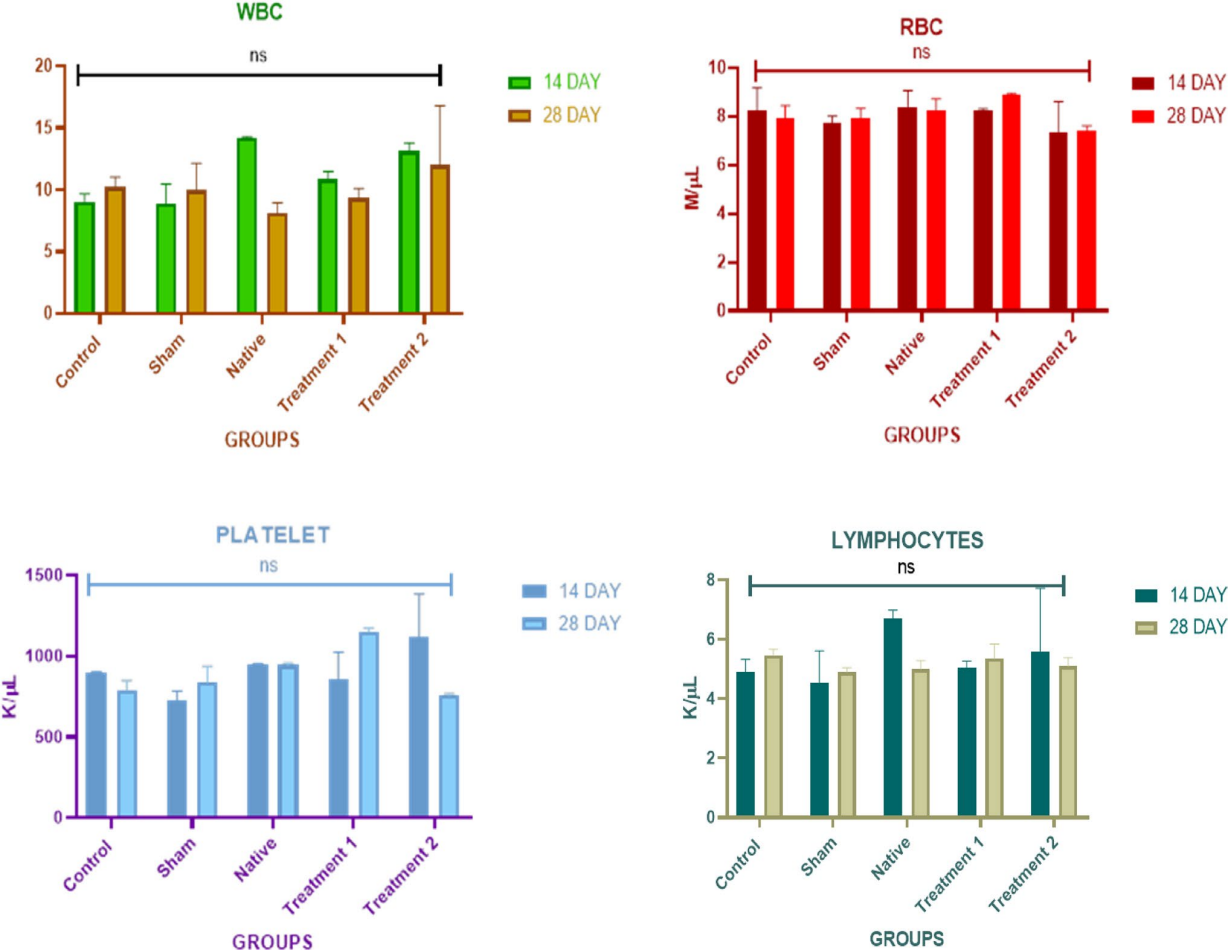
Blood count analyses (**A**) White blood cell count, (**B**) Red blood cell count, (**C**) Platelet count, and (**D**) Lymphocyte count. Measurements were taken on days 14 and 28 for all groups. (ns-non significant).

The hematological parameters of Wistar rats implanted with native and DTFS were analysed on days 14 and 28 to evaluate systemic inflammatory and immunological responses. As in Fig. 7, no statistically significant differences were observed across groups for white blood cell (WBC), red blood cell (RBC), platelet count, or lymphocyte levels at either time point (*p* > 0.05). The WBC Count of native and treatment 2 showed slight increase at day 14, but all values remained within the physiological range (Fig. 7A). The RBC levels remained stable across all groups, with no significant deviations on either day 14 or 28 (Fig. 7B). Minor increase in platelet was observed in the Treatment 1 group by day 28, although not statistically significant (Fig. 7C), while lymphocyte levels fluctuated slightly across all groups but stayed within baseline reference ranges (Fig. 7D).

### Serum urea levels

**Fig. 8:**
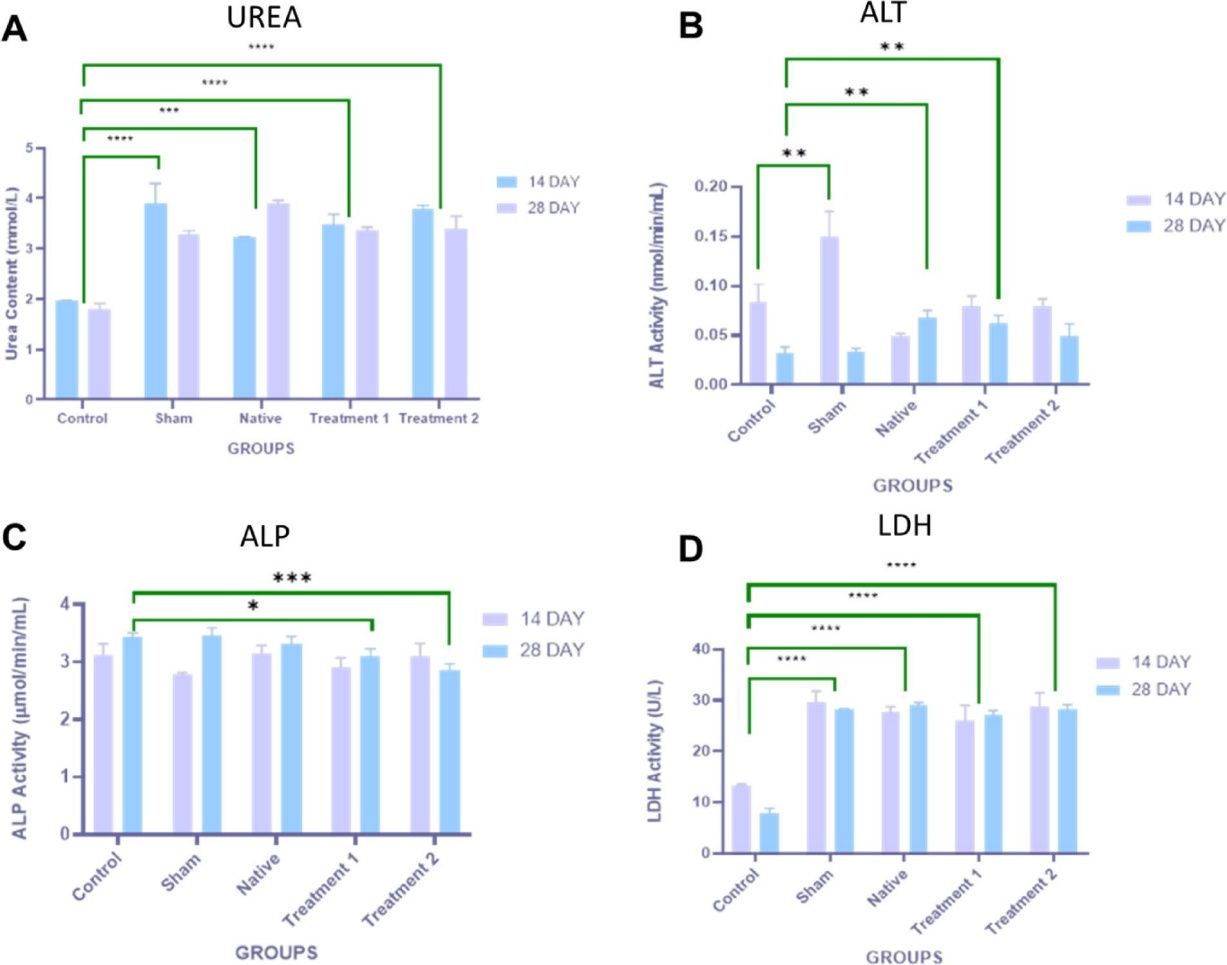
Biochemical tests in Wistar rats at days 14 and 28 post-implantation. (*p < 0.05.**p < 0.01, ***p < 0.001, ****p < 0.0001) (**A**) Urea content (mmol/L) (**B**) Serum ALT activity (mmol/min/mL) (**C**) Serum alkaline phosphatase (ALP) activity (µmol/min/mL) (**D**) Serum LDH activity (U/L).

Serum urea concentration was measured to assess renal function and systemic biocompatibility of the implanted native and DTFS. As shown in Fig. 8A, the Sham group exhibited significantly elevated urea levels on day 14 (*p* < 0.0001) compared to others. In contrast, urea levels in the Native, Treatment 1, and Treatment 2 groups were comparable to the Control group at both day 14 and day 28 (*p* > 0.05), indicating no signs of renal stress or toxicity from implantation. The urea levels in DTFS groups remained stable between both time points, further confirming the absence of adverse metabolic or systemic responses.

### Alanine transaminase (ALT) activity

Serum ALT activity was evaluated to assess hepatic function and detect potential hepatocellular injury following implantation. As shown in Fig. 8B, a significant elevation in ALT levels was observed in the Sham group on day 14 compared to the others (*p* < 0.01). ALT levels in the Treatment 1 & 2 groups remained low and statistically comparable to the control across both day 14 and day 28. In contrast, the Sham group, which underwent surgical incision without implantation, exhibited a short-term spike in ALT on day 14 that normalized by day 28. These findings suggest that DTFS do not induce hepatocellular toxicity and maintain liver enzyme homeostasis during subcutaneous implantation in vivo.

### Alkaline phosphatase (ALP) activity

Serum ALP activity was evaluated to investigate hepatic and biliary responses to implantation. As shown in Fig. 8C, ALP levels in the Sham group were significantly elevated on day 14 compared to the Control group (*p* < 0.05) and to the Treatment 1 & 2 groups (*p* < 0.001). The Native group also showed a modest increase in ALP activity at day 14, although levels returned to baseline by day 28. In contrast, DTFS groups-maintained ALP activity within the normal physiological range throughout the study period. No statistically significant variation was observed between any group at either time point.

### Lactate dehydrogenase (LDH) activity

LDH activity was assessed to evaluate cellular membrane integrity and potential tissue damage associated with implantation. As shown in Fig. 8D, a significant elevation in LDH activity was observed in the sham and native groups on day 14 compared to the Control group (*p* < 0.0001). These elevated levels suggest early cellular stress or injury. In contrast, Treatment 1 & 2 maintained LDH activity within physiological limits, statistically comparable to the Control group at both time points (*p* > 0.05). There was no significant difference in LDH levels between day 14 and 28 in any of the groups, indicating stable systemic responses throughout the study duration.

### Histological examination of rat skin

**Fig. 9:**
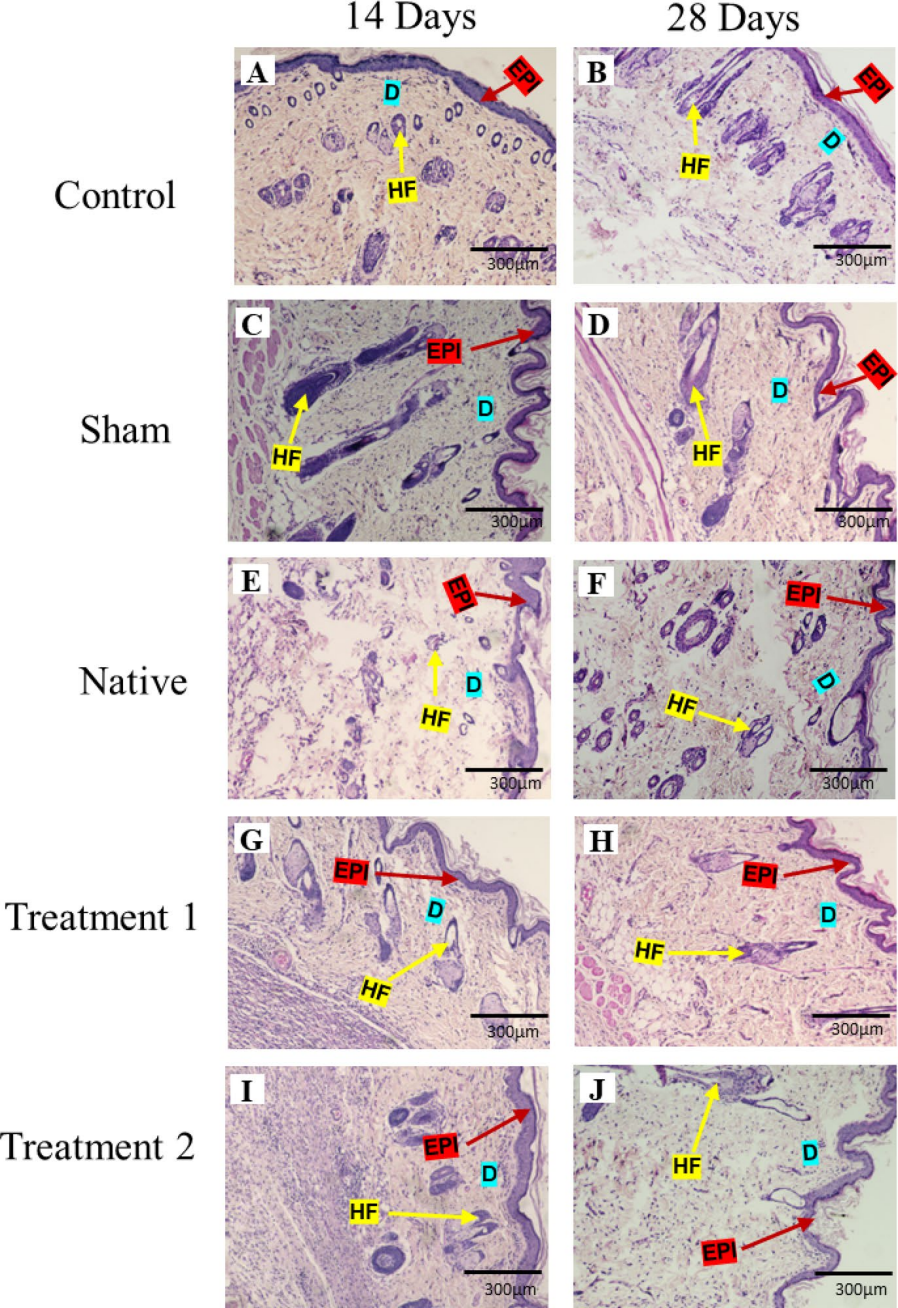

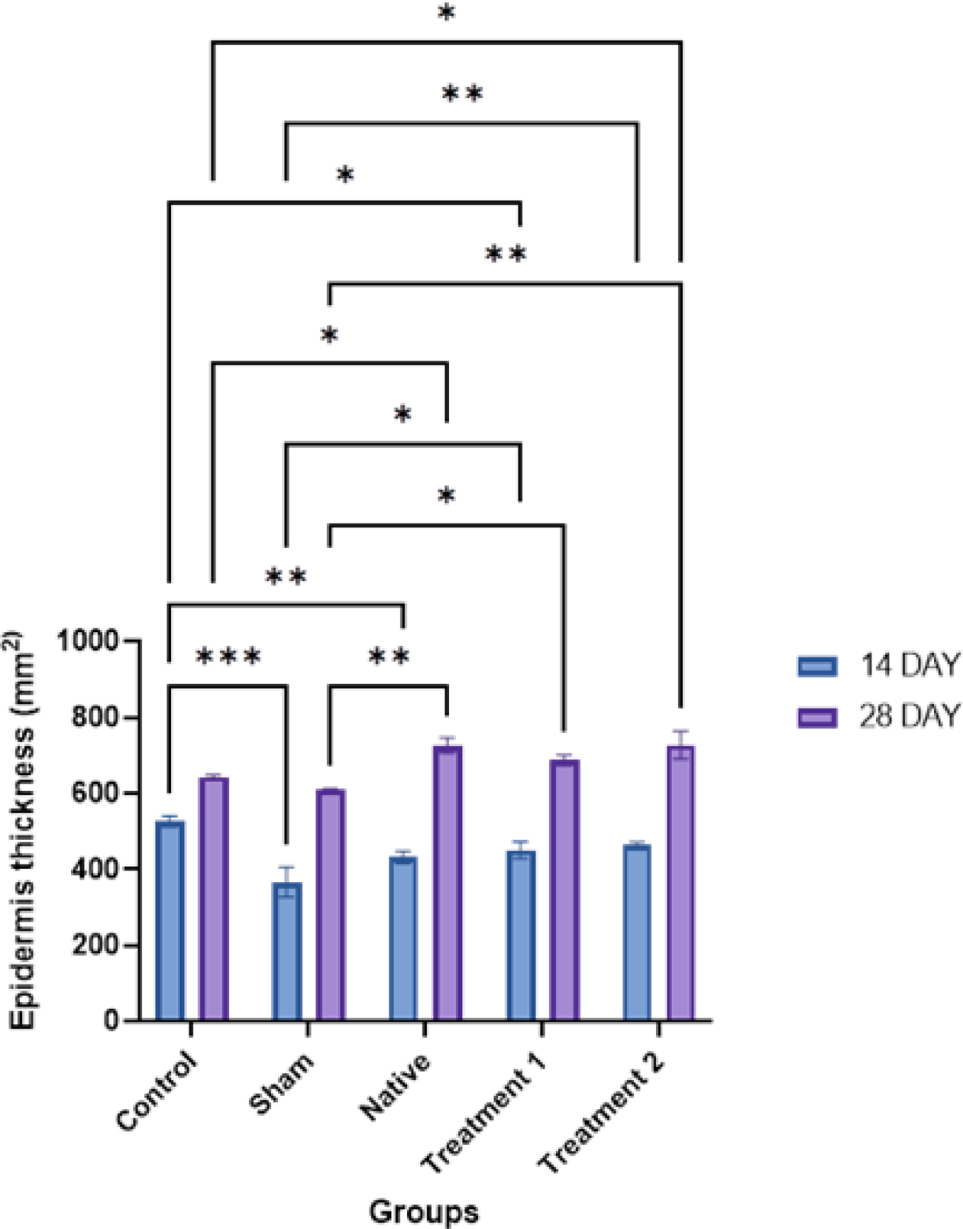
(i) Hematoxylin and eosin staining of rat skin in 10X magnification on days 14 and 28 post-implantation. EPI indicates epidermis (Red arrow), D indicates Dermis (blue colour), and HF indicates hair follicles (Yellow arrow). Control are untreated rats; Sham rats are subjected to incision and suturing; Native (rats implanted with a tilapia fish skin), Treatment 1 (rats implanted with DTFS as per treatment 1), and Treatment 2 (rats implanted with DTFS as per treatment 2). (ii) Histomorphological analysis of rat skin at the implantation site. Epidermal thickness is measured using ImageJ software. (*p < 0.05.**p < 0.01, ***p < 0.001, ****p < 0.0001).

Histological analysis of rat skin tissues was performed 28 days post-implantation using H&E staining to evaluate tissue response, inflammation, and morphological changes. As illustrated in Fig. 9(i), the Control group (A&B) exhibited normal skin architecture, with a well-defined epidermis and dermis, intact hair follicles, and no inflammatory infiltration. The sham group (C&D), which underwent surgical incision and suturing, displayed mild inflammatory cell infiltration in the dermis, consistent with surgical trauma and wound healing. The native group (E&F) showed signs of disorganized collagen arrangement and mild edema, likely due to residual antigenic components in the non-decellularized. In contrast, Treatment 1 (G&H) & 2 (I&J) demonstrated well-organized skin structure with restored epidermal integrity, absence of inflammatory infiltrates, and normal dermal morphology. No signs of fibrosis, necrosis, or granuloma formation were observed, indicating good tissue integration.

### Histomorphic analysis

Epidermal thickness was assessed at 14 and 28 days post-treatment (Fig. 9[ii]). At day 14, compared to the control, the sham group exhibited the lowest; Treatment 1 & 2 groups displayed greater epidermal thickness. By day 28, epidermal thickness increased across all groups. The sham group remained lower than the control. Notably, Treatment 1 and Treatment 2 showed the highest epidermal thickness. Statistical analysis confirmed significant differences between groups, with Treatment 1 and Treatment 2 showing marked improvements compared to the sham and native. The progressive increase from day 14 to day 28 highlights enhanced epidermal regeneration in treated groups.

### Histological examination of rat liver

**Fig. 10:**
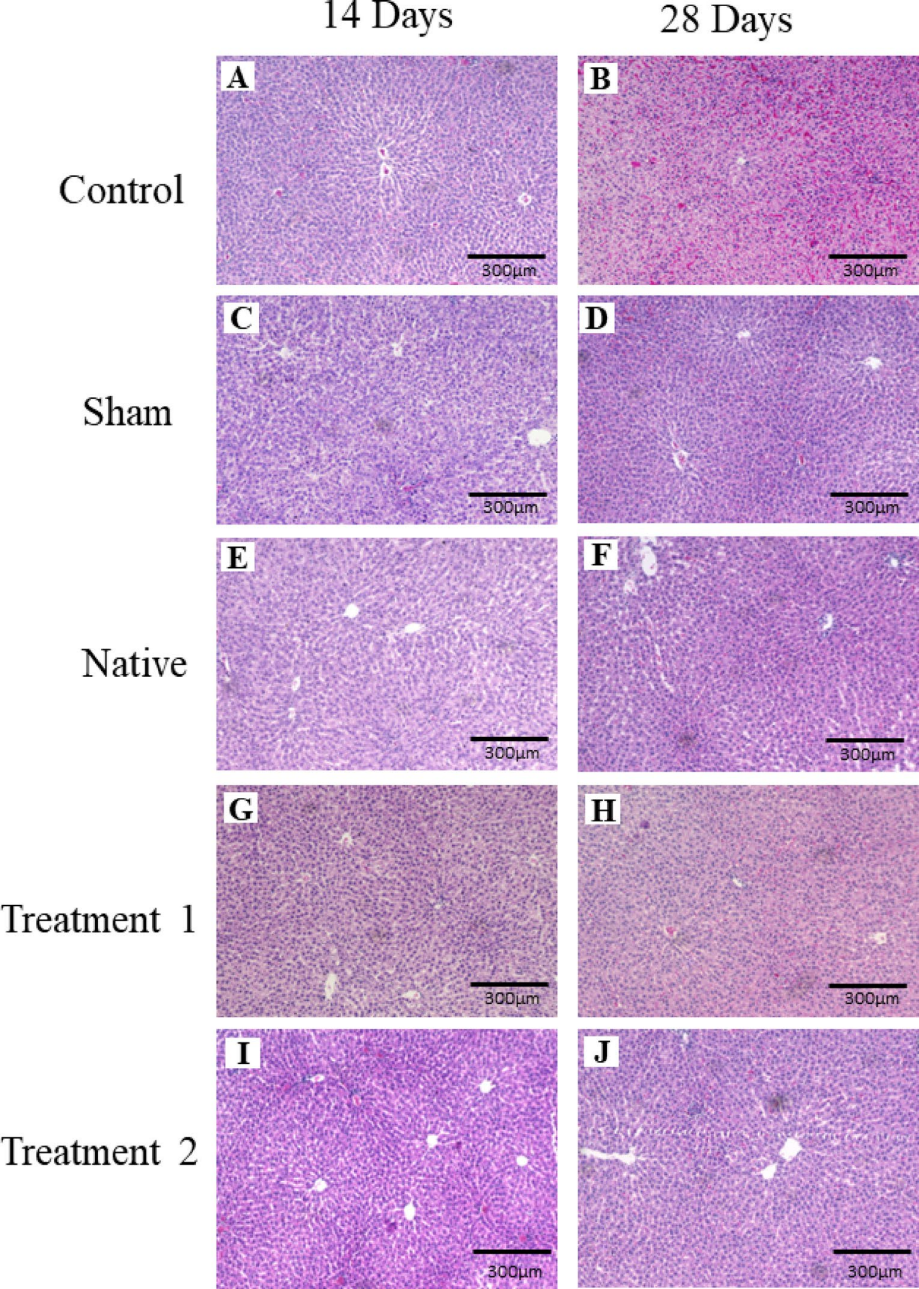
Hematoxylin and eosin staining of rat liver in 10X magnification on days 14 and 28 post-implantation. Control are untreated rats; Sham rats are subjected to incision and suturing; Native (rats implanted with a tilapia fish skin), Treatment 1 (rats implanted with DTFS as per treatment 1), and Treatment 2 (rats implanted with DTFS as per treatment 2).

Histopathological analysis of liver tissues was performed on days 14 & 28 post-implantation to evaluate systemic biocompatibility and detect any hepatocellular toxicity. As shown in Fig. 10, liver sections from the control group(A&B) (at both time points) displayed normal lobular architecture, central vein clarity, intact hepatocyte morphology, and absence of inflammatory infiltration. In contrast, the sham group(C&D) (particularly day 14) exhibited signs of mild hepatocellular degeneration and focal inflammatory infiltration, indicative of transient surgical stress. The native group(E&F) also showed mild sinusoidal congestion and patchy hepatocyte swelling, suggestive of a localized immune response due to xenogeneic antigen presence. However, liver sections from both DTFS groups, Treatment 1(G&H) and Treatment 2 (I&J) at day 14 and day 28 demonstrated preserved hepatic architecture, with no evidence of necrosis, fibrosis, or immune cell infiltration. The hepatocytes appeared normal, and sinusoids were intact, indicating the absence of material-induced systemic toxicity.

## Discussion

The present study comprehensively investigated the decellularization efficacy, structural integrity, and in vivo biocompatibility of tilapia fish skin processed using two distinct chemical protocols. Both treatments effectively reduced residual DNA content to levels below the accepted immunogenic threshold^26^, with Treatment 1 achieving superior nucleic acid clearance. Importantly, ECM architecture was preserved post-decellularization, as evidenced by histological and ultrastructural analyses^27^. In vivo biocompatibility studies in Wistar rats demonstrated no significant perturbations in hematological or biochemical markers, and histological assessments of skin and liver tissues confirmed minimal inflammatory responses and absence of systemic toxicity. Compared to native, DTFS showed markedly enhanced host integration and immune tolerance. Collectively, these findings underscore the translational promise of DTFS as a low-cost, biocompatible implant for soft tissue engineering and wound healing applications, particularly in resource-constrained healthcare environments. In Fig. 2D, we have reported 10 different decellularization techniques, which did not lead to effective decellularization when compared to treatments 1& 2.

### Effectiveness of decellularization methods based on DNA content

Efficient decellularization is a critical prerequisite for minimizing host immune response and ensuring implant biocompatibility^28^. Regulatory standards, including those proposed by Crapo et al. (2011) (12), suggest that residual DNA content should ideally remain below 50 ng/mg dry tissue to qualify as decellularized material. In this study, treatment 1&2 showed reduced DNA content well below the threshold, with treatment 1 achieving the lowest DNA content (8.65 ± 1.25 ng/mg), followed by treatment 2 (18.05 ± 0.85 ng/mg). The observed difference between the two methods may be attributed to the ionic strength of SDS, a known anionic detergent that disrupts nuclear membranes and facilitates DNA release^29^, compared to the milder non-ionic action of Triton X-100. Moreover, the inclusion of NaCl in Treatment 1 may enhance osmotic lysis^30^, further contributing to cellular material clearance. These findings are consistent with prior studies demonstrating that SDS-based protocols tend to yield more complete decellularization^31^. Agarose gel electrophoresis demonstrated DNA removal efficacy, indicating a clear smear for native tissue and an absence of DNA bands in DTFS. This suggests both protocols sufficiently degraded or extracted nucleic acids to levels that are unlikely to provoke inflammatory or immunogenic responses upon implantation^32^. The Table 2 summarizes the treatment methods available for DTFS, highlighting the variation in chemical composition, which is different in this current study. Overall, this study validates treatment 1&2 protocol of decellularization as novel and unique from what is reported in literature.

**Table 2:** Scoring of rat skin on a scale of (0–3), 0 - absent; 1- mild; 2 - moderate; 3 – severe.

Parameters	Control (14 days)	Control (28 days)	Sham (14 days)	Sham (28 days)	Native (14 days)	Native (28 days)	Treatment 1 (14 days)	Treatment 1 (28 days)	Treatment 2 (14 days)	Treatment 2 (28 days)
Epithelialization of epidermis	3	3	3	3	3	2	3	3	3	3
Rete ridge	3	3	2	3	1	2	2	3	2	3
Hair follicles	3	3	2	2	1	3	1	1	1	1
Adipose cells in the dermis	2	2	1	1	2	2	1	2	1	1
Fibroblast in the dermis	2	2	2	2	2	2	2	2	2	2
Inflammatory cells in the dermis	1	1	1	1	1	1	1	1	1	1
Collagen deposition in dermis	3	3	3	3	3	3	3	3	3	3

### Structural preservation and decellularization efficiency by H&E

Histological evaluation through H&E staining provides a qualitative assessment of the decellularization efficacy and ECM preservation^33^. In this study, DTFS (Treatment 1 & 2) resulted in complete removal of cellular and nuclear material, as evidenced by the absence of hematoxylin-stained nuclei. This is a critical criterion for biocompatible implant preparation, as residual nuclear material may be associated with immunogenicity and poor host integration^34^. The preservation of the collagenous architecture in DTFS further supports the functional integrity of the ECM post-treatment. The characteristic crisscross collagen pattern observed in native fish skin was retained in DTFS, which is essential for maintaining mechanical properties, porosity, and cell-instructive cues necessary for tissue regeneration^35^. Thus both treatment 1&2 were equally effective in cellular clearance with minimal ECM damage, which also aligns with literature. This ECM-retaining, non-immunogenic implant are potential in wound healing, cartilage regeneration, and skin graft applications making DTFS a cost-effective and biocompatible candidate for clinical translation.

### Microstructural evaluation of native and decellularized implants

SEM analysis provided essential insights into the microstructural remodeling induced by different decellularization methods^36^. Native tilapia skin displayed a tightly packed, stratified morphology consistent with unprocessed biological dermis, as also reported in prior fish skin studies. This compactness reflects the presence of intact cellular material and dense collagen lamellae, which are critical for tensile strength but may impede cellular infiltration if left untreated^37^. DTFS of treatment 1 retained a moderately compact structure with partial porosity, indicative of cellular removal while largely preserving ECM fiber alignment. In contrast, Treatment 2 led to more pronounced ECM delamination and fiber unraveling. This may be attributed to the lower protein denaturation potential of Triton X, which allows for ECM preservation but may promote excessive loosening at extended exposure. The resulting porous architecture may be advantageous for cell seeding and nutrient diffusion for tissue-engineered constructs^38^.

### SDS page

The SDS-PAGE analysis of native and DTFS revealed the preservation of key collagen components, particularly the α1 and α2 chains at ~ 135 kDa and 180 kDa, the β chain at ~ 245 kDa. The presence of these characteristic collagen bands in both native and DTFS suggests that the decellularization process successfully retained the ECM architecture. Since collagen type I is the major structural protein responsible for providing mechanical strength and biological functionality to the ECM, its preservation is a critical indicator of scaffold integrity. The reduction of non-collagenous protein bands in DTFS samples further confirms the effective removal of cellular materials, which is essential for minimizing immunogenicity in biomaterial applications. Importantly, the comparable banding patterns between native and DTFS demonstrate that the decellularization protocol did not disrupt the primary structural framework of collagen. These findings are consistent with previous studies^39^ on decellularized fish and mammalian tissues, where retention of collagen integrity was reported as a prerequisite for their use in regenerative medicine and tissue engineering.

### Cell viability assay

The cell viability assay demonstrated that both native and DTFS were highly biocompatible, as reflected by the consistently high cell survival rates across tested concentrations. The observation of nearly 100% viability with native at 6 µg, and similarly high viability with DTFS (Treatment 1 and 2) at lower concentrations (2 µg and 0.7 µg), suggests that the decellularization process did not introduce cytotoxic by-products, demonstrating cytocompatibility. These results are consistent with literature^40^ on decellularized biological scaffolds, where retention of ECM components, particularly collagen, provides a favorable microenvironment that supports cell attachment and proliferation. The high cell viability also indicates that DTFS may elicit minimal to no immune or inflammatory responses in vitro, further reinforcing its suitability as a biomaterial.

### Systemic Immunohematological evaluation

Hematological parameters are sensitive indicators of systemic inflammation and immune activation following biomaterial implantation. In this study, the absence of significant deviations in WBC, RBC, platelet, and lymphocyte counts in DTFS indicates that neither protocol elicited overt immune reactions or hematologic toxicity in vivo. The slight elevation of WBCs in the native group at day 14 may be attributed to the presence of residual cellular components, consistent with previous findings where non-decellularized implant prompted a modest leukocytic response^27^. In contrast, both treatment 1&2 maintained WBC counts within normal limits, further validating the efficacy of the decellularization protocols (Fig. 7A). Platelet count elevation in the treatment 1 group at day 28, could suggest platelet recruitment linked to early wound healing processes. However, these remained within the physiological range and were not associated with elevated WBCs or lymphocytes, ruling out thrombotic or inflammatory pathology. Lymphocyte levels across all groups showed no significant deviation, suggesting a lack of lymphocyte-driven adaptive immune activation, which supports the immune compatibility of fish-derived collagen and ECM components. RBC stability across all groups rules out hemolytic or marrow-suppressive effects of the implant. These findings align with earlier studies where decellularized ECM-based scaffolds including marine-derived biomaterials exhibited minimal systemic immune activation, promoting their candidacy for clinical translation^41^.

### Renal biocompatibility and Urea homeostasis

Serum urea is a key metabolic indicator of nitrogen excretion and kidney function, while elevated levels may signal impaired renal clearance, catabolic stress, or systemic toxicity. In this study, the sham group showed a significant transient increase in serum urea at day 14 as shown in (Fig. 8A). This may reflect post-surgical metabolic stress or localized inflammatory response due to mechanical injury, which tends to resolve with tissue recovery. Importantly, DTFS demonstrated urea concentrations comparable to the control, indicating no nephrotoxic or immunometabolic disturbances over the 28-day evaluation period. These findings support the in vivo biocompatibility of the DTFS, affirming its safety for systemic applications. Preservation of urea levels within normal physiological ranges aligns with previous preclinical studies on marine collagen scaffolds^42^, reinforcing the low immunogenicity and metabolic inertness of fish-derived ECM materials. By maintaining biochemical homeostasis in implanted animals, these DTFS show potential for clinical translation in burn therapy, wound healing, and tissue engineering without systemic side effects.

### ALT as a marker of hepatic safety

Alanine transaminase (ALT) is a well-established biomarker for liver injury, with elevated levels reflecting hepatocyte membrane disruption or inflammatory responses. In the context of biomaterial evaluation, ALT measurement is crucial to ensure that implant degradation products or immune responses do not exert hepatotoxic effects^43^. The significant rise in ALT levels observed in sham on day 14 likely results from tissue stress or transient inflammation associated with surgical trauma, consistent with previous reports in rodent models^44^ as shown in (Fig. 8B). The return to baseline by day 28 confirms spontaneous hepatic recovery without chronic toxicity. In contrast, DTFS demonstrated ALT levels within normal physiological ranges at both time points. This indicates that neither the SDS-NaCl nor the Triton X-100–NaOH treatment protocols elicited systemic toxicity or hepatic dysfunction. These results are in line with earlier studies on decellularized fish-derived scaffolds, which have shown excellent systemic biocompatibility and minimal off-target organ impact. Importantly, the absence of ALT elevation in DTFS reinforces the safety profile of tilapia skin-based biomaterials and supports their application in long-term subdermal or topical therapeutic contexts, such as wound healing and dermal repair^45^.

### ALP as a marker of hepatobiliary integrity

Alkaline phosphatase (ALP) is a membrane-bound enzyme predominantly associated with hepatobiliary and bone activity. Elevated ALP levels may indicate hepatic inflammation, biliary obstruction, or membrane stress induced by systemic toxicity or xenogeneic scaffold response. In this study (Fig. 8C), the sham group exhibited a significant spike in ALP activity on day 14, likely a transient hepatic reaction to surgical trauma, which resolved by day 28. This is consistent with prior reports where minor surgical interventions alone induced short-term hepatobiliary enzyme elevations^46^. The Native group showed slightly elevated ALP levels, potentially due to residual cellular debris or immunogenic factors inherent to non-decellularized fish skin. However, the most critical finding is that both DTFS did not induce ALP elevation, indicating no hepatic or biliary stress post-implantation. These findings affirm that decellularization significantly improves scaffold safety by eliminating antigenic components, reducing the risk of hepatobiliary toxicity. Similar outcomes have been reported in other marine collagen and fish skin scaffold studies, further supporting the non-toxic and biocompatible nature of these ECM-based materials for clinical use^47^.

### LDH as a marker of tissue integrity and biocompatibility

Lactate dehydrogenase (LDH) is a cytoplasmic enzyme released into the bloodstream upon cell membrane damage or necrosis. It serves as a sensitive biomarker for cytotoxicity, inflammation, and tissue injury making it a reliable tool in scaffold biocompatibility evaluations. Significantly increased LDH activity in the sham group at day 14 is attributed to surgical stress and post-incision tissue inflammation as shown in (Fig. 8D). Similarly, the native group showed elevated LDH levels, likely due to residual immunogenic components, such as cellular debris and unprocessed proteins, consistent with earlier reports where non-decellularized xenografts induced systemic stress responses^48^. Conversely, DTFS displayed LDH values consistent with the control, indicates that detergent protocol prevented systemic cytotoxicity or membrane damage. These results reinforce the earlier findings from ALT and ALP assays, supporting the overall non-toxic and biocompatible nature of the DTFS. The retention of LDH activity within normal ranges suggests minimal inflammatory or necrotic responses, validating the implant suitability for in vivo applications. These outcomes are also aligned with prior in vivo studies on marine-derived scaffolds, which demonstrate excellent tolerance and integration without inducing systemic stress^49^.

### Histocompatibility and skin regeneration

Histological analysis is a gold standard for assessing local tissue compatibility and scaffold-host interactions in vivo. In this study, skin sections from rats implanted with DTFS exhibited excellent integration with host tissue, characterized by a lack of chronic inflammation, re-epithelialization, and normal dermal architecture. The mild inflammation observed in the sham group is consistent with typical surgical wound healing phases and resolved by day 28, as seen in similar implantation models^50^. In the native group, disorganized dermis and mild edema suggest an immune response to residual cellular content, a common issue in xenogeneic grafts without proper decellularization. Scoring of skin on various parameters, including epithelization of epidermis, rete ridge, hair follicles, adipose, fibroblast and inflammatory cells in dermis and collagen deposition in dermis, are reported on a scale of 0–3 as shown in Table 2, (0 - absent; 1- mild; 2 - moderate; 3 – severe). DTFS, led to histologically normal skin regeneration, supporting the biocompatibility and regenerative capacity of these implants. Prior studies have shown that ECM scaffolds retaining their native collagen architecture, while being devoid of antigenic cellular material, promote angiogenesis, tissue remodelling, and accelerated healing^51^. These outcomes reinforce earlier biochemical and haematological findings, further validating DTFS as a safe, histocompatible, and effective biomaterial for soft tissue repair.

### Hepatic histocompatibility and systemic safety

The liver, being a primary site of metabolic processing and immune response, serves as a critical indicator of systemic toxicity and scaffold biodegradation impact. Histological analysis in this study revealed that DTFS did not induce hepatocellular damage, confirming their hepatic safety profile. Mild hepatocyte degeneration and inflammation in the sham group at day 14 likely resulted from surgical trauma, consistent with transient liver stress observed in similar rodent models^52^. The native group, showed mild hepatic changes, aligns with earlier findings that non-decellularized xenografts can trigger systemic immune responses due to residual cellular antigens. In contrast, both treatment 1 and 2 groups maintained normal hepatic morphology with no pathological alterations across both time points. Inflammatory cells, hepatocellular necrosis, and liver fibrosis were assessed using a scoring system ranging from 0 to 4. The scale is defined by 0 reporting no hepatocellular necrosis, edema, inflammatory cell infiltration, or fibrosis, while 1 indicates Mild fibrous portal expansion, and 2 represent moderate bridging fibrosis, 3 shows severe bridging fibrosis with architectural distortion, 4 indicates Liver cirrhosis. The control, sham, native, treatment 1, and treatment 2 groups all received a score of 0 for each of these parameters, indicating no liver damage. These observations are strongly supported by the ALT and ALP biochemical profiles, which also remained within physiological ranges for the decellularized groups. The preservation of hepatic structure, absence of inflammatory infiltration, and maintenance of vascular integrity further validate the biocompatibility and systemic safety of these decellularized implants. These results are consistent with prior studies using fish-derived biomaterials for regenerative medicine applications^53^, which reinforces the clinical potential of tilapia skin scaffolds.

## Conclusion

This DTFS, processed via optimized SDS-NaCl and Triton X-100–NaOH protocols, as a structurally robust, biocompatible, and non-toxic, which will be suitable for diverse biomedical applications. Both treatment strategies demonstrated efficient removal of immunogenic cellular components while preserving the architectural and functional integrity of ECM, as evidenced by comprehensive biochemical, histological, and ultrastructural analyses. In vivo evaluations u on Wistar rat confirmed systemic biocompatibility, with no significant perturbations in haematological parameters, liver and renal function biomarkers, or tissue histology. Importantly, DTFS exhibited superior host integration and immunological tolerance compared to native fish skin, underscoring their potential as safe and effective regenerative biomaterials.

These findings reinforce the translational promise of DTFS, an underutilized marine byproduct. This work aligns with the United Nations Sustainable Development Goals [SDG 3 (Good Health and Well-Being), SDG 12 (Responsible Consumption and Production), and SDG 9 (Industry, Innovation and Infrastructure)]. Taken together, DTFS represents a scalable and socially impactful platform for next-generation wound healing and tissue regeneration therapies.

## Prospects

DTFS offers a promising, low-cost alternative to conventional wound dressings, especially in burn management and chronic ulcers. In India, clinical translation of such xenogeneic biological dressings requires adherence to the Medical Devices Rules, 2017 under the Central Drugs Standard Control Organization (CDSCO). DTFS, when intended for therapeutic use, would be classified as a Class C or D medical device, necessitating: Biocompatibility and toxicity testing as per ISO 10,993, Sterilization validation under ISO 11,137, Preclinical (animal) studies, Institutional Ethics Committee (IEC) approval, Application for Form MD-15 (manufacture) or MD-41 (import license). The recent release of regulatory guidance for tissue-engineered products and regenerative biomaterials by CDSCO and ICMR has streamlined pathways for novel biological scaffolds, including decellularized marine tissues.

## Data Availability

The authors confirm that the data supporting the findings of this study are available within the article.
